# Role of the KEAP1-NRF2 Axis in Renal Cell Carcinoma

**DOI:** 10.3390/cancers12113458

**Published:** 2020-11-20

**Authors:** Sara Clerici, Alessandra Boletta

**Affiliations:** IRCCS San Raffaele Scientific Institute, Molecular Basis of Cystic Kidney Diseases, Division of Genetics and Cell Biology, 20132 Milan, Italy; clerici.sara@hsr.it

**Keywords:** NRF2, KEAP1, ccRCC, PRCC, kidney injury

## Abstract

**Simple Summary:**

Renal cancers are common types of tumors affecting humans. While many different genes have been found to be mutated and to drive the initiation and progression of these lethal cancers, a fine molecular understanding of the process is still lacking. One important pathway that emerges central in many different types of renal cancers is one called KEAP-NRF2. This axis is very important in normal kidneys as a defense against oxidative stress. Here, we summarize a large body of literature suggesting that this axis is exploited by tumor cells to escape control and to transform, and thus it could represent a good target for therapy.

**Abstract:**

NRF2 is a transcription factor that coordinates the antioxidant response in many different tissues, ensuring cytoprotection from endogenous and exogenous stress stimuli. In the kidney, its function is essential in appropriate cellular response to oxidative stress, however its aberrant activation supports progression, metastasis, and resistance to therapies in renal cell carcinoma, similarly to what happens in other nonrenal cancers. While at the moment direct inhibitors of NRF2 are not available, understanding the molecular mechanisms that regulate its hyperactivation in specific tumor types is crucial as it may open new therapeutic perspectives. Here, we focus our attention on renal cell carcinoma, describing how NRF2 hyperactivation can contribute to tumor progression and chemoresistance. Furthermore, we highlight the mechanism whereby the many pathways that are generally altered in these tumors converge to dysregulation of the KEAP1-NRF2 axis.

## 1. Introduction

**Figure 1:**
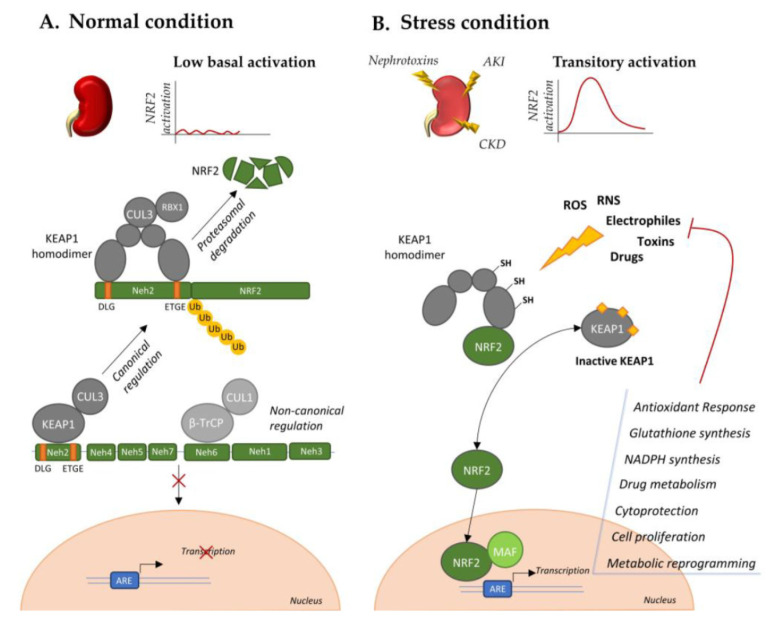
NRF2 regulation in normal and stress conditions. (**A**). NRF2 activity in normal condition is maintained at low levels through KEAP1-dependent canonical ubiquitination and KEAP1-independent noncanonical pattern. (**B**). Stress stimuli, through electrophilic binding of KEAP1, induce its conformational modification and release of NRF2. NRF2 nuclear translocation transiently activates, through MAF binding, the transcription of a signature aimed to counteract the initial stress.

The nuclear factor erythroid 2 (NFE)-related factor 2 (NRF2) is encoded by the *NFE2L2* gene and is member of cap ‘n’ collar (CNC) subfamily of basic leucine zipper (bZip) transcription factors. It comprises seven functional domains (Neh1–7) for the binding with regulatory proteins; coactivators and regulatory sequences on target genes. Indeed, NRF2 translocates to the nucleus after binding with small musculo-aponeurotic fibrosarcoma (sMAF) protein to CNC in Neh1 domain and activates the transcription of target genes through the recognition of antioxidant responsive element (ARE) in their promoter [1,2]. NRF2 is mainly involved in the activation of antioxidant response [3] consequently to stress stimuli, promoting the production of proteins and enzymes suitable for the reduction of reactive oxygen species (ROS) and reactive nitrogen species (RNS) in specific compartments of the cell [4]. Thus, among the principal targets of NRF2 there are genes involved in biogenesis of reducing factors and regeneration of their oxidized forms, as glutathione (GSH) and thioredoxin (TRX), stress responsive proteins, as heme oxygenase (HO)-1, and proteins involved in catabolism of superoxides and peroxides [4]. Moreover, NRF2 signature comprises genes belonging to different cytoprotective pathways, drug metabolism, and disposition pattern, as NAD(P)H:quinone oxidoreductase 1 (NQO1), proteasomal protein degradation, cell proliferation and metabolic reprogramming [4]. In normal conditions, NRF2 is maintained at low levels by the activity of its negative regulator Kelch-like ECH-associated protein 1 (KEAP1) [5]. It recognizes the Neh2 domain of NRF2, in particular the DLG and ETGE motifs allowing the formation of a heterodimer with E3 ubiquitin ligase cullin 3 (CUL3), thus promoting NRF2 degradation. However, in stress conditions, it functions as a sensor able to initiate NRF2 response through its release. In fact, the covalent binding of electrophilic activators to KEAP1 cysteine residues impairs KEAP1-dependent ubiquitination of NRF2, allowing its nuclear translocation and transcription of target genes. This mechanism, in concert with other noncanonical regulatory pathway later discussed in this review, accounts for transitory activation of NRF2 when the cells are subjected to endogenous or exogenous insults in stress or pathological conditions [1]. This is particularly relevant in kidney tissue, in which pathological conditions (such as hyperglycemia and hypertension) or exogenous toxic stimuli (such as nephrotoxins and chemotherapies) can induce kidney injury through a detrimental ROS accumulation, while low levels of ROS maintain kidney homeostasis (Figure 1). Their uncontrolled accumulation accounts for oxidative stress that represents an aggravating factor for initiation and progression of both acute (AKI) and chronic (CKD) kidney injury. Nowadays, there are no approved treatments for these pathologies, with the patients undertaking dialysis and renal transplant according to the disease progression [6,7]. Thus, a transitory activation of NRF2 and its downstream antioxidant signature represents a promising strategy for renal pathologies, with pharmacological activators under investigation [8]. For example, Bordoxolone methyl, which displayed contrasting results in the treatment of diabetic nephropathies [7], is currently in clinical trial for the treatment of autosomal dominant polycystic kidney disease (ADPKD) (*NCT03918447*). Indeed, impairment of NRF2 antioxidant activity has been described as a driver of ADPKD, and its pharmacological activation accounts for reduced cystogenesis and progression in an orthologue model of the disease [9]. Although activators of NRF2 are opening new perspectives in the treatment of different renal pathologies, underlying its protective role in kidney tissue, its aberrant hyperactivation is becoming a central driver of progression of different cancer types, such as renal cell carcinoma. Unfortunately, the production of NRF2 direct inhibitors is a complex and unresolved issue in the oncologic field, thus, understanding the KEAP1-NRF2 axis role and regulation in different cancer types can provide interesting alternative therapeutic approaches.

RCC is a worldwide disease, with high incidence and mortality. Only in 2018, about 400,000 new cases and 175,000 deaths were registered (Global Cancer Observatory). RCC per se accounts for more than 90% of kidney cancers [10]. However, in recent years, the concept that RCC is not a single entity arose, leading to the characterization of a panel of cancer subtypes featuring different histology, clinical course, and response to therapy [11]. The main classification is based on histological analysis. The two major groups are defined as clear cell RCC (ccRCC), which represents about 75% of RCC, and nonclear cell RCC (nccRCC). In this second category, papillary renal cell carcinoma (pRCC) represents approximately 15% of all RCC, while chromophobe RCC accounts for 5%. Further analysis led to identification of two variants of pRCC: type I, with basophilic cytoplasm, and type II, with eosinophilic one [10,11]. The remaining RCC cases are more rare subtypes, defined based on mutations, cell of origin, or structure [10]. Adding an additional layer of complexity in the study of RCC, each histological subtype shows a proper genetic and molecular signature. Recent comprehensive multidimensional evidence allowed to distinguish the alterations that seem to be specific for different cancers, allowing for identification of different subclasses. This characterization is critical for the design of patient management, diagnostic tools, and therapies. In accordance, The Cancer Genome Atlas (TCGA) developed three projects in order to map genetic and epigenetic signatures of the three major histological RCC subtypes: KIRC (kidney renal clear cell carcinoma) [12], KIRP (kidney renal papillary cell carcinoma) [13], and KICH (kidney chromophobe) [14]. These studies, together with comparative analysis developed among RCC histological subtypes, highlighted chromosomal alterations, cancer metabolic reprogramming, and immune expression profiles that are specific for each, or shared among the principal histological RCC subgroups [14,15]. This led to the identification of a pRCC with CpG island methylator phenotype (CIMP-RCC) [14,15], and a metabolically divergent RCC in the chromophobe subgroup [14]. Indeed, several pathways potentially involved in RCC subtypes progression were found to be altered by somatic mutations, genomic rearrangements, or epigenetic modifications targeting newly found or already known key genes.

ccRCC is mainly characterized by genomic alteration in the short arm of chromosome 3 that encodes for the Von Hippel Lindau tumor suppressor gene *VHL*, reported in almost 90% of the cases [16]. Only the 2–3% of ccRCC is hereditary, while somatic mutations, hypermethylation, or deletion occurs in the majority of the cases [16]. In normoxic conditions, VHL participates in polyubiquitination and proteasomal degradation of hypoxia-inducible factor HIF, that is stabilized as a consequence of VHL degradation occurring when oxygen levels drop. Loss of VHL stabilizes HIF1α and HIF2α in normal oxygen conditions, leading to a state of pseudohypoxia. Thus, the activation of HIF-dependent transcription of genes such as *VEGF*, *TGF-β*, and glycolytic enzymes supports a protumorigenic adaptation, which results in enhanced angiogenesis, cell proliferation, and metabolic rewiring (Warburg effect) [17]. ccRCCs that are not mutated in *VHL* carry mutations mainly in the PI3K/AKT/mTOR pathway, with an upregulation of mTORC1 signaling observed in about 80% of the cases, in the chromatin remodeling and histone modifying pathways. 

pRCC is an heterogenous subtype of kidney cancer, characterized by the common feature of papillae formation. As already mentioned, PRCC can be discriminated in type I and type II subgroups that, besides a peculiar histological feature, show distinct mutational and metabolic profiles. Type I pRCC features frequent concurrent gains in chromosomes 7 and 17, which encode for potential oncogenes, such as *BRAF*, *EGFR*, and *MET*. Among them, activating mutations or alterations in the *MET* signature have been reported in 81% of cases, with a prevalence of somatic or germline mutations in the *MET* gene (18.6%), which results in the upregulation of cell survival, proliferation, and migration pathways. Type II pRCC can occur both sporadically and associated to hereditary leiomyomatosis and renal cell carcinoma (HLRCC). While sporadic type II pRCC mainly displays mutations in cyclin-dependent kinase inhibitor 2A (*CDKN2A*), histone lysine methyltransferase (*SETD2*), and transcription factor E3 (*TFE3*) [13,18], hereditary type II pRCC is triggered by germ line mutations in the fumarate hydratase gene (*FH*), an enzyme of the tricarboxylic acid cycle (TCA) [19], which causes an intracellular accumulation of the oncometabolite fumarate. Moreover, CpG Island Methylator Phenotype-RCC (CIMP-RCC) has been recently identified by TCGA analysis as a new nonsyndromic subtype of aggressive, early onset, high-stage pRCC [13], associated with the poorest survival among all the RCC histological subgroups [14]. This group of tumors features both somatic and germline mutations in the *FH* gene, resulting in fumarate accumulation, as described for HLRCC patients [11,13,14]. Interestingly, CIMP-RCC shows a peculiar metabolic profile among pRCC subtypes, with a strong decrease in Krebs cycle genes and increase in the signature of ribose metabolism [11]. This metabolic rewiring supports tumor growth and proliferation through the production of ribose sugars, and counteracts the intracellular stress produced by fumarate accumulation through supplement of NADPH and regeneration of glutathione (GSH) [20,21,22].

## 2. NRF2 Expression in PRCC and ccRCC

TheNRF2 pathway has been recently reported to be altered in 4.7% of all RCCs analyzed, both in ccRCC and pRCC, with a prevalence in CIMP-RCC [14,15]. The NRF2-ARE signature, which was initially characterized in PRCCs, has been recently reported to be altered in 3.2% of ccRCCs [14], becoming one of the molecular alterations that manifests transversally among RCC histological subtypes. Mutations involving the major players of the NRF2 pathway are generally mutually exclusive, even if they can co-occur in tumors with known association to exposure to carcinogenic factors [23,24]. These mutations involve directly the NRF2 coding gene *NFE2L2* [25,26], but also genes encoding for the regulatory proteins KEAP1 [26] and CUL3 [23], and NRF2 transcriptional targets (Table 1). Moreover, the promoter of the *KEAP1* gene is frequently methylated specifically in ccRCC, resulting in downregulation of its mRNA, although it remains controversial whether or not expression of the protein is also altered [27,28]. These genetic and epigenetic modification, along with other post-translational modifications that will be further discussed in this review, account for a frequent observation of the upregulation and activation of the NRF2 gene and protein, as well as upregulated transcription of its principal target genes [25,29]. Interestingly, despite the well-assessed cytoprotective role in normal kidney, the chronic activation of the NRF2 pathway in ccRCC supports tumor progression and metastasis formation [26,29]. Indeed, the expression levels of NRF2 and its pathway associates with cancer grading and staging, and poor prognosis, impairing the postoperative renal function and the response to therapeutic agents [25,26,27,28,29].

The NRF2 pathway is hyperactivated in almost all types of pRCC at different grades. Multimolecular analysis reported high activation in CIMP-RCC, medium in type II pRCC, and low in type I pRCC [15,30]. Despite activating hotspots in *NFE2L2* and inactivating mutations in its negative regulators, KEAP1, CUL3, and SIRT1 have been reported, they do not justify themselves the overexpression of NRF2 transcriptional signature in the different tumor types. Indeed, NRF2 activity can be modulated through post-translational modifications that target directly NRF2, or its interacting proteins. In particular, fumarate, that accumulates in *FH*-deficient tumors as type II CIMP-RCC and HLRCC, can directly succinate both KEAP1 and DJ-1 modulating NRF2, with a different impact on tumor growth and survival [20,31,32]. However, as described for ccRCC, hyperactivation of the NRF2 signature associates with tumor progression and decreased survival [13].

## 3. NRF Hyperactivation Supports RCC

### 3.1. Balancing Cytoprotection and Chemoresistance

Among the principal roles of the KEAP1-NRF2 axis there is sensing of oxidative stress and activation of a transitory cytoprotective response. This occurs in normal tissues, when KEAP1 cysteine residues are modified by ROS accumulation, allowing NRF2 nuclear translocation and transcription of target genes, as players of the antioxidant response, drug metabolizing enzymes, efflux pumps, and transporters. The response implies the activation of metabolic pathways that ensure the production of molecules such as GSH, a tripeptide thiol antioxidant that acts as a cysteine reservoir and ROS scavenger, whose reduction strictly depends on NADPH availability [33]. This NRF2-dependent mechanism is central in protecting normal tissues from both endogenous and exogenous oxidative stress, caused for example by carcinogenic compounds or radio- and chemotherapy. However, different studies and reviews have pointed out that a chronic hyperactivation of NRF2 signature transforms a cytoprotective pattern into a protumorigenic one, leading to the definition of “the dark side” of NRF2 [34]. Cancers that feature chronic activation of NRF2 signature, such as RCC, are highly proliferative and metastatic, and show metabolic advantage and resistance to therapies. Different models have been proposed to explain the contradictory role of NRF2 in normal and malignant cells. One suggests that NRF2 activates a different transcription signature in the two contexts [35]. Another pointed out that ARE domains, that are bound by NRF2 to initiate transcription, are different comparing tumor and normal cells, with the cancer-ARE localized where chromatin is more accessible than noncancer-ARE. Indeed, the regulation of NRF2 can be tissue specific and differ between normal and malignant cells [36]. Moreover, as reported above, the timing of NRF2 activation is crucial to understand its role: in cancers, it is chronically hyperactivated, whereas in normal tissue it is constantly repressed by KEAP1-dependent degradation and activated only in response to stress stimuli. This is the case of kidney cancers, where transitory activation of the NRF2 signature protects the kidney epithelium from carcinogens and chemotherapies such as cisplatin, while its hyperactivation in ccRCC and PRCC is crucial in supporting a malignant phenotype and chemoresistance [36,37].

Cisplatin is a common chemotherapeutic drug for solid tumors, able to induce DNA adducts formation and oxidative stress. However, its main limitation is a marked nephrotoxicity, occurring as impaired renal function and induced apoptosis and necrosis in the kidney epithelium. NRF2 plays a crucial role in protecting the kidney epithelium from cisplatin treatment, supported by the fact that *Nrf2* null mice feature a higher nephrotoxicity [37,38]. However, cisplatin itself, inducing ROS accumulation, activates NRF2-dependent production of cytoprotective genes and transporters in the kidney epithelium [37,38]. Indeed, NRF2 activators were reported to reduce cisplatin cytotoxicity in kidney epithelial cells, thus allowing to think about a combinatory treatment to prevent chemotherapy side effects, even if their effect is still contradictory in in vivo experiments because of biodistribution and bioavailability [37,38,39,40,41]. Moreover, the combination between cytotoxic and antioxidant anti-inflammatory compounds is able to counteract chemically induced ccRCC formation through NFR2 activation [42]. In a similar way, both ccRCC and PRCC exploit the chronic hyperactivation of NRF2 and transcription of antioxidant genes and drug metabolizing enzymes to overcome radio- and chemotherapy-induced cytotoxicity. Advanced metastatic RCC displays a poor response to excision and drug resistance, which can develop also in response to target therapies, such as Sunitinib. However, downregulation of NRF2, not only impairs RCC cells viability, invasion, and migration, but promotes cell cycle arrest and apoptosis after Sunitib treatment [43]. The same was reported in relation to Axatinib, a second generation of VEGF inhibitor, and As2O3, that is approved for the treatment of some kind of leukemia, which impair RCC viability and whose activity is counteracted by hyperactivation of NRF2 [28,44]. There are several mechanisms that sustain NRF2 hyperactivation and chemoresistance in RCC and, as the downstream signature, can vary among tissues and differ between malignant and normal cells. Thus, understanding how the principal players of this cascade are modified and interact with each other can provide interesting perspectives for the treatment of this heterogenous type of cancer.

### 3.2. Epithelial-to-Mesenchymal Transition

As previously described, the NRF2 hyperactivation that characterizes tumors as RCCs not only supports tumor growth and survival, but prompts toward a malignant phenotype, with increased metastasizing capacity [25,26,44]. Epithelial-to-mesenchymal transition (EMT) is a dynamic and reversible mechanism, in which epithelial cells feature the reactivation of embryonic program through induction of specific transcription factors (such as Zeb1, Twist, Snail, and Slug) that ensure downregulation of epithelial markers, such as E-cadherin, and increase in mesenchymal ones, such as N-cadherin. As a result, cells acquire a migratory and malignant potential that supports metastasis formation. This occurs also in metastatic RCC, where the EMT process is activated by different mechanisms, such as chronic oxidative stress, loss of VHL, and stabilization of HIF-1 α and activation of Wilm’s tumor transcription factor 1, that induces an epithelial-to-mesenchymal hybrid transition in which the cells retain both epithelial and mesenchymal features [45]. NRF2 plays a controversial role in relation to the EMT process, depending on the tissue in which the transformation occurs. In cancer tissue, NRF2 activation supports the EMT process and drug resistance. It impairs E-cadherin expression and its inhibition accounts for reduction in N-cadherin and metalloproteases production [46]. In addition, its activation sustains the crosstalk between tumor and inflammatory cells through paracrine mechanisms, coordinating the shift toward M2 proinflammatory phenotype in macrophages and the EMT process in liver and pancreas tumor cells [47]. Indeed, cancers harboring mutations in the *NFE2L2* gene and featuring NRF2 constitutive activation show increased proliferation, anchorage-independent growth, and metastatic potential, dependent on mTORC1 activation. Moreover, expression of mutant *NRF2* gene in human embryonic kidney (HEK)-293 cells is sufficient to confer them an oncogenic and metastatic phenotype [48]. Curiously, it was suggested that NRF2 prevents a complete transition supporting the induction of both E-cadherin and Zeb-1 at the same time. As a consequence, NRF2 activation maintains the cancer cell in a hybrid state that is strongly associated with more aggressive metastatic potential in different cancer types, as in RCC [49,50]. Moreover, cells displaying a hybrid epithelial/mesenchymal phenotype are more prone to develop drug resistance [51], a mechanism highly supported by NRF2 hyperactivation. Consistently, transforming growth factor (TGF) β1, a known driver of EMT process, induces NRF2 stabilization in a p21-dependent manner in transformed cells of squamous cell carcinoma, in which it sustains radio- and chemo-resistance through the regulation of glutathione metabolism [52]. The fact that a transformed phenotype confers drug resistance through NRF2 activation is further supported by the observation that E-cadherin downregulation induces NRF2 stabilization in hepatoma cells, conferring chemoresistance [53]. Thus, it is plausible that NRF2 could drive a similar mechanism in RCCs featuring its hyperactivation. Interestingly, NRF2 opposes the EMT process in different nontransformed tissues, especially the kidney. In normal tissue, EMT is activated not only during organogenesis and development, but also in response to organ damage [54]. It was reported to drive kidney fibrosis through the trans-differentiation of tubular epithelial cells into collagen-producing ones similar to myofibroblasts [55,56]. Kidney fibrosis is a common feature of diabetic nephropathy, in which TGFβ1 activation and ROS accumulation supports EMT-dependent fibrosis, which results in loss of functionality and insurgence of CKD. This mechanism is counteracted by a transitory activation of NRF2 and its downstream antioxidant signature, as *HMOX1* and genes involved in GSH biosynthesis. Indeed, NRF2 activators, such as Sulfurophane, Epigallocatechin-3-gallate, and curcumin, represent a good option for the treatment of acute and chronic kidney disease [57,58,59,60,61]. This further supports the idea that NRF2 role can vary depending on the tissue and the extent of its activation, thus investigating the differential interactors and mechanisms of regulation can be useful to point out new therapeutic approaches. 

## 4. NRF2 Regulation in RCC

### 4.1. Mutations in Genes that Directly or Indirectly Affect KEAP1-NRF2

In order to understand how genetic alterations in the KEAP1-NRF2 axis can impact on cancer progression in the kidney, it is helpful to describe the major animal models that feature NRF2 hyperactivation with implications in this tissue. *Keap1* knockout mice feature postnatal lethality, due to hyperkeratosis in the upper digestive tract, which is corrected by local deletion of the *Nrf2* gene [62]. Interestingly, these mice show polyuria and kidney damage, probably due to downregulation of aquaporin 2 and reduced water resorption, as demonstrated in mice with *Keap1* specific deletion in renal tubules. This suggests a role of NRF2 in regulating cell fate and organism homeostasis, other than the already described cytoprotective and detoxifying activity [63,64]. Indeed, it allows to think that a persistent activation of the NRF2 pathway could have a detrimental role in some tissues, compared to an oscillatory activation in response to stress stimuli, as previously described. This is particularly relevant in tumors as RCC, in which the NRF2 signature is often hyperactivated compared to the normal tissue and supports tumor progression. However, systemic or kidney-specific deletion of the *Keap1* gene is not sufficient to cause an aggressive tumor formation, suggesting that NRF2 plays a role in supporting tumor growth and drug resistance, but not in tumorigenesis [64]. This is further sustained by the observation that human germline loss of function mutations in the *KEAP1* gene are not associated with cancer formation, even if it predisposes to multinodular goiters [65].

While in cancers featuring FH downregulation (such as HLRCC) the accumulation of fumarate is the main cause of NRF2 hyperactivation through the post-translational modification of its regulatory proteins [20,66], NRF2 signature regulation in sporadic forms of RCCs is more complex. Several mutations involve directly the *NFE2L2* gene and are highly conserved among different cancer types [24,67]. The analysis of TCGA catalogue has identified almost 2% of unique NRF2-mutant tumors among all cases reported, with the 63% of tumor type harboring *NLE2L2* mutations [24]. These mutations localize mainly in ETGE and DLG motives of the *Neh2* domain, which determine KEAP1 ability to bind NRF2 and direct its CUL3-dependent degradation. This results in the stabilization of the NRF2 protein and upregulation of its transcriptional activity [24,48]. In RCCs, *NFE2L2* is among the 20 aberrant genes, harboring mainly missense mutations in activating hotspots, prevalently reported in PRCC [13,15] (Table 2). Interestingly, almost all these mutations are predicted to be subclonal and converging on a single gene in the same pathway, suggesting that they are subjected to a strong selection [68]. However, NRF2 signature harbors mutations also in 3.2% of ccRCCs [14], with 2% of mutations involving directly *NFE2L2* [68] and copy number alteration in position 2q31.2 [69] (Table 1). Indeed, rs6721961 single nucleotide polymorphism (SNP) in the promoter of *NFE2L2* gene has been described to support carcinogenesis [70,71]. However, *NFE2L2* gene alterations in the primary tumor, both in homo- and in heterozygosity, impact not only on tumor progression, but also on the clinical outcome and in the response to therapy, with patients developing chronic kidney disease after partial nephrectomy [25] and reduced response of metastasis to vascular endothelial growth factor-targeting therapy [26]. However, the described *NFE2L2* alterations are not the only cause of the hyperactivation of NRF2-ARE signatures reported in RCCs. Indeed, a plethora of mutations involving genes encoding for the regulatory interactors of NRF2 have been reported in different types of RCC (Table 1). Mutations in *KEAP1* and *CUL3* genes are mutually exclusive with the one in the *NFE2L2* gene present in 6.6% of ccRCCs; indeed 10.4% of the tumor analyzed presented a deletion in the *CUL3* locus 2q36 [23]. Inactivating mutations in the same genes are described also in PRCCs [13,72]. In addition, a dominant negative mutation in the *SIRT1* gene has been described in co-occurance with *NFE2L2* mutation in one case of sporadic type II PRCC. This impairs SIRT1 deacetylase activity, resulting in increased NRF2 nuclear translocation and consequent transcriptional activity [72]. This is in line with several studies underlying the involvement of class III histone deacetylases in multiple processes of cancer initiation and progression and associating SIRT1 downregulation in RCCs with a poor prognosis [73,74,75].

### 4.2. Epigenetic Regulation of KEAP1-NRF2 Axis

Mutations in the key genes of the NRF2 pathway are not always sufficient to justify its aberrantly increased transcriptional activity in different solid tumors, such as lung cancer and PRCC [13,79]. The NRF2 axis is subjected to epigenetic regulation, which is the most frequent mechanism of downregulation of *KEAP1* in solid tumors, caused by the methylation of CpGs located in the P1 region of the promoter, near the transcriptional starting site [80]. This is relevant in RCC, since DNA methylation and epigenetic modifications have been extensively investigated in this set of tumors, resulting in the characterization of a new type II PRCC (CIMP-PRCC) associated with hypermethylation of CpG islands [13]. However, Fabrizio et al. demonstrated that the hypermethylation of the *KEAP1* promoter is peculiar of ccRCCs compared to PRCC, with an incidence of 49% in the samples analyzed. The observation was validated through two datasets of ccRCC and PRCC from the TCGA portal, that outlined a strong correlation between *KEAP1* promoter hypermethylation and ccRCC staging, grading, and overall survival [27]. Indeed, it is known that also the *NFE2L2* promoter is subjected to epigenetic modifications in some kinds of cancer. In particular, it was reported that 5-fluorouracyl, through the production of ROS, activates DNA demethylases that act on the *NFE2L2* promoter and result in increased NRF2 transcription. Colorectal cancer featuring hypomethylation of the *NFE2L2* promoter and consequent hyperactivation of the NRF2 axis shows resistance to different chemotherapeutic drugs [81,82,83]. Even if the epigenetic regulation of the *NFE2L2* promoter has not yet been investigated in RCC, understanding the balance between its hypomethylation and the hypermethylation of the *KEAP1* promoter can be informative in the development of new therapeutic strategies.

**Figure 2:**
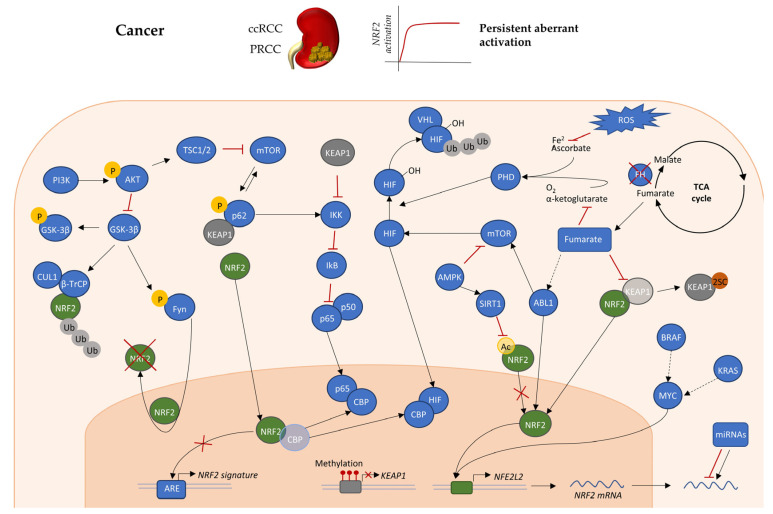
KEAP1-NRF2 modulation in cancers such as RCCs. The main pathways involved in KEAP1-NRF2 are epigenetic, transcriptional, and post-translational regulation in cancer. These pathways are generally mutated and deregulated in ccRCC or PRCC, thus they can account for the aberrant and persistent NRF2 activation observed in these tumor types. Dashed arrows indicate an indirect link between the two interactors of the pathway. Post-translational modifications are indicated as phosphorylation (P), ubiquitination (Ub), acetylation (Ac), and succination (2SC). Red dots on *KEAP1* promoter indicate methylations.

Among the mechanisms of epigenetic regulation there are miRNAs, responsible for downregulation of target mRNA translation through base-paired binding of the 3′-UTR. Several miRNAs have been reported to target the NRF2-ARE axis (Figure 2). Some act directly on *NFE2L2*, accounting for the downregulation of transcription of NRF2 target genes in both tumors, such as miR-144 [84], and normal tissue, such as miR-28 [85]. Another subset of miRNAs targets *KEAP1* and other crossing points of the NRF2 network, resulting in the upregulation of the NRF2 axis that characterize RCCs. Indeed, meta-analysis on human RCC allowed to identify eight key miRNAs associated with the phenotype [86]. Among the most significant, there is the class of miR-200, containing five members that are classified as epithelial markers and suppressors of epithelial-to-mesenchymal transition (EMT) [87,88], a process tightly regulated by NRF2 in kidney tissue [45]. These miRNAs are mainly downregulated in ccRCC, especially in metastatic tissue as compared to primary tumors, supporting their possible involvement in the regulation of the metastasis process [89]. In particular, miR-200b and miR-200c downregulation associates with metastasis formation and poor prognosis in ccRCC patients [90,91]. Indeed, miR-200c, through the downregulation of *HMOX1*, a target gene of NRF2, sensitizes ccRCC to chemotherapeutic agents [92], suggesting that miRNAs regulation of the NRF2-ARE signature can impact on different features of RCC. Indeed, miR-200a has been reported as a negative regulator of KEAP1, indirectly increasing NRF2 levels in breast and liver cancer [34,93,94]. Hypoxia-responding miR-101 downregulates *Cul3*, which is directly involved in NRF2 ubiquitination, thus accounting for the upregulation of its signature. This mechanism supports the induction of VEGF, HO-1, and eNOS, that induce KEAP1 nytrosylation leading to a positive feedback loop on NRF2 [95]. Curiously, miR-101 is downregulated in ccRCC and the consequent overexpression of its targets (e.g., DONSON) associates with resistance to Sunitinib treatment, while its restoration inhibits the invasive behavior of RCC cells [96,97]. Moreover, the downregulation of miR-32 found in RCC is considered as a marker of poor prognosis and has been proposed to be indirectly linked to the upregulation of NRF2 observed in RCCs, since in prostate cancer it downregulates PI3K, a known negative regulator of NRF2 [98]. Thus, even if a precise definition of which specific miRNAs impact on the NRF2-ARE signature in the different types of RCC is lacking, multiple lines of evidence suggest that they can play a crucial role in supporting NRF2 aberrant activation in cancer.

### 4.3. Oncogenes and Transcriptional Regulation of KEAP1-NRF2 Axis

Regulation of NRF2 transcription is far less studied as compared to mechanisms that act at the post-transcriptional level. DeNicola et al. demonstrated that specific oncogenes directly induce the upregulation of NRF2 mRNA. In particular, the expression of endogenous K-RAS, B-RAF, or MYC increases NRF2 mRNA and transcription of target genes that account for reduction in intracellular ROS (Figure 2). In particular, the authors demonstrated that the *NFE2L2* gene presents a site for the direct binding of Myc. They further showed that K-RAS and B-RAF induction of NRF2 transcription depends on MYC and JUN. Curiously, the activation of an axis responsible for ROS-detoxification here is shown to contribute to tumor progression, while in other tumors it is the ROS accumulation to trigger carcinogenesis, as previously underlined. This can be attributed to the role of other NRF2-target genes, as drug metabolizing enzymes, growth factors, and receptors, or to the persistent activation of NRF2, which has been described to support a malignant phenotype [35]. Among the oncogenes highlighted as regulators of the NRF2 axis, MYC is described to play a key role in RCC. Genomic amplifications are reported in 5–10% of ccRCC patients, with overexpression in 20% of cases [12], that account for the activation of the *MYC* pathway in most human RCCs [99]. Indeed, activation of the MYC oncogene, but not Ras, is reported to play a primary role in initiating and maintaining RCC in transgenic mice [100]. On the other hand, despite the fact that amplification of KRAS was described in RCC, as reported for c-MYC [101], mutations in RAS and BRAF are extremely rare in kidney tumors (almost 1%) [102,103,104]. While some studies outlined a different incidence of mutations among the RAS isoforms in RCC samples, with 0–16% of KRAS mutation and very rare events in NRAS and HRAS [105], others did not detect any mutation of KRAS, such as BRAF, neither in primary nor in metastatic ccRCC or PRCC [106]. However, mutations are not the only mechanism accounting for oncogenes activation, thus the low rate of mutation of some oncogenes in RCC does not exclude that they can play a crucial role in progression of these types of tumors. Indeed, it has been demonstrated that activated H-RAS, through a kinases cascade involving RAF and extracellular signal-regulated kinase (ERK), promotes NRF2 nuclear translocation and an increase in HO-1, which results in protection of renal cancer cells from apoptosis [107].

A particular mechanism of regulation regards the interplay between NRF2 and NF-κB. The two pathways tightly co-operate in the maintenance of cellular homeostasis and response to stress stimuli. In fact, *Nrf2* null mice develop a neurodegenerative phenotype, in which the progressive demyelination is triggered by NF-κB-dependent cytokines production [108,109]. NRF2 control of NF-κB activity plays a crucial role in different pathologies featuring chronic inflammation. In lupus nephritis, the increased NRF2 activation leads to GSH accumulation that reduces the levels of free radicals and NF-κB-dependent inflammatory response [110]. The reduction of NRF2 during aging accounts for the exacerbation of the renal phenotype and establishment of the chronic inflammatory status [111]. This mechanism of regulation is supported also by KEAP1, which, other than regulating NRF2 degradation, is involved in inhibition of the NF-κB pathway [112,113]. However, this kind of balance between NRF2 and the NF-κB pathway is maintained through a bidirectional regulation; indeed, p65, one of the principal players in NF-κB canonical pathway, accounts for activation or inhibition of NRF2, depending on the environment and the cell type. p65 was reported to repress NRF2 signaling through a direct interaction and nuclear accumulation of KEAP1 [114], or competing with NRF2 for the binding of histone acetyltransferase CREB binding protein (CBP)-p300, that supports transcription through histone acetylation and regulation of non-histone substrates, as NF-κB and NRF2 [115] (Figure 2). However, in acute myeloid leukemia NF-κB has an opposite effect on NRF2, that presents several κB sites in its proximal promoter. In this context, p65 directly induces *NRF2* transcription and overexpression, accounting for resistance to chemotherapy [116]. Thus, it is possible that NF-κB transcriptional regulation of NRF2 plays a crucial role in cancers where the two axes are concomitantly hyperactivated. This is the case of RCC, especially in ccRCC which features loss of VHL, a NF-κB negative regulator [117,118]. In this context, the nuclear accumulation of p65 [119,120] and NF-κB hyperactivation contributes to tumor progression and chemoresistance [121].

Interestingly, *NRF2* promoter, in addition to κB sites, contains ARE-like elements. Thus, the overexpression of NRF2 can activate a positive feedback loop that directly supports its transcription and nuclear accumulation, representing another level of transcriptional regulation [122].

### 4.4. Post-Translational Modifications Affecting the KEAP1-NRF2 Axis

In addition to the canonical mechanism of regulation, involving KEAP-1-dependent degradation, NRF2 is directly subjected to post-translational modifications that influence its subcellular localization and stabilization. Indeed, NRF2 contains many serine, threonine, and tyrosine residues which provide sites for phosphorylation by different pathways involving kinases, such as ERK and mitogen-activated protein kinase cascades (MAPK), protein kinase C (PKC), and phosphatidylinositol 3-kinase (PI3K/AKT)/glycogen synthase kinase 3-beta (GSK-3β) [123].

The PI3K/AKT pathway plays a central role in RCC, where it is reported to be highly mutated and represents one of the predominant targets of the FDA-approved drugs for these kinds of tumors [124]. Curiously, co-occurrence between mutations in PI3K/AKT and NRF2 pathways have been reported in different kind of tumors [125], and activation of PI3K was described to induce NRF2 accumulation and metabolic rewiring with the aim to support cell proliferation and protection from oxidative stress [126], suggesting a direct interaction among the two pathways. A similar pattern of regulation has been described in both transformed renal adenocarcinoma cells and normal renal tubular epithelial cells, where insulin, through the activation of PI3K/Akt pathway, induces NRF2 phosphorylation, nuclear translocation, and production of HO-1. The PI3K/Akt-dependent induction of NRF2 signature has been reported to be independent of both PKC [127] and ERK and p38-MAPK cascade, whose role is extremely controversial depending on the model employed for the studies [128]. Moreover, PI3K/AKT can promote NRF2 nuclear localization and stability through the inhibitory phosphorylation of GSK-3β (Figure 2). Indeed, GSK-3β coordinates both the KEAP1-independent degradation of NRF2 through phosphorylation of serine residues in the Neh6 domain, subsequently recognized and ubiquitinylated by SCF/β-TrCP-mediated complex [129], and the NRF2 nuclear export, and cytoplasmic accumulation [130,131,132], through Fyn-mediated phosphorylation [131]. GSK-3β-dependent regulation of the NRF2 axis is central in the response of normal epithelial kidney tissue to stress stimuli. In tubules featuring acute kidney injury (AKI), induced for example by radiotherapy, hyperactivation of GSK-3β results in impaired NRF2 nuclear accumulation, mitigated induction of antioxidant genes, and consequent oxidative damage, which accounts for the insurgency of chronic kidney disease [133]. In this context, a transitory activation of the NRF2 axis in response to stress is beneficial for the normal tissue, again supporting the idea that the NRF2 role in promoting a malignant phenotype depends on its chronic hyperactivation. In fact, treatment with salvianolic acid, through AKT activation and consequent GSK-3β inhibition, induces NRF2 nuclear accumulation and transcription of target genes that protect the kidney epithelium from the oxidative stress produced during chronic kidney disease (CKD) [134]. Curiously, GSK-3β is generally overexpressed in RCC, where it supports tumor cells proliferation and it has been pointed out as a promising target for RCC treatment, with 9-ING-41 inhibitor in clinical trial [135], partially contrasting with the NRF2 accumulation and contribution to RCC progression.

However, the role of NRF2 post-translational modification in promoting its nuclear accumulation and protumorigenic activity in RCC has been reported by different studies. Recently, Yu et al. described that BMP8A is overexpressed in ccRCC and promotes proliferation, metastasis, and drug resistance through Nrf2 activity. In particular, they propose a model in which increased phosphorylation of NRF2 interferes with KEAP1-dependent degradation, allowing its nuclear translocation and transcription of *TRIM24*, that through activation of the WNT pathway supports RCC progression [44].

In addition to kinases that directly modify players of the KEAP1-NRF2 axis, there are components able to destabilize their interaction and causing a different activation of downstream signaling pathways. We have already described that p65, for example, can directly bind KEAP1 or CBP-p300 in order to suppress NRF2 signaling. On the other hand, p62, a stress-inducible protein acting as ubiquitin-binding autophagy receptor, has been described as a KEAP1-binding protein capable of supporting the hyperactivation of NRF2 signature, especially in cancer (Figure 2). The regulation between p62 and NRF2 axis is bidirectional, as described for p65. p62 is encoded by the *SQSTM1* gene on chromosome 5, which presents ARE domains through which NRF2 can induce p62 transcription in response to oxidative stress [136]. Furthermore, a positive feedback loop, whereby p62 sequesters KEAP1 and triggers its autophagic degradation, thus sustaining NRF2 activation, can also be involved. This mechanism relies on the direct binding between the p62 KIR domain and the KEAP1 DC one. When p62 is overexpressed or phosphorylated on Ser349, it can display KEAP1 DC domain binding with NRF2 DLG domain, releasing NRF2 for nuclear translocation [136,137]. This post-translational modification depends on kinases that in normal tissue are activated in response to stress stimuli, while in cancer these are generally mutated or hyperactivated. This is the case of mTOR, which is highly mutated in ccRCC [14], where it plays a role in the regulation of the p62-NRF2 balance at different levels (Figure 2). Indeed, other than phosphorylating p62 promoting KEAP1 sequestration [138], its inhibition in RCC cells induces both p62 and NRF2 but prevents its nuclear translocation through GSK-3β activation [139], further supporting mTOR involvement in NRF2 activation in RCC. The impact of this mechanism of regulation varies among different tumor types. For example, it was demonstrated that the hyperactivation of mTORC1 and p62 triggers renal carcinogenesis in models of tuberous sclerosis characterized by inactivation of the tuberous sclerosis complex 2 (TSC2), but in this case it does not rely on the consequent hyperactivation of Nrf2 [140,141]. On the other hand, ccRCC features copy number gains on chromosome 5q, which contains the *SQSTM1* gene, in 70% of the cases with consequent overexpression of p62. Even if the KEAP1-binding domain of p62 was shown to be neither necessary nor sufficient to promote renal transformation, the amplification of the *SQSTM1* gene is mutually exclusive with the far less frequently reported mutations in the *NRF2* gene. This suggests that NRF2 is particularly relevant for the role of p62 in RCC, but it acts in concert with other p62 substrates to support cancer progression, more than tumorigenesis [142]. The observation that this regulation depends on the cancer type, is further supported by the fact that p62 upregulation is central in promoting hepatocellular carcinoma (HCC) formation and resistance to therapies specifically through NRF2 activation [143,144]. Indeed, specific inhibitors targeting the interaction between P-p62 and Keap1, opposing tumor cells proliferation and chemoresistance have been proposed as treatment for HCC, and could be exploited also in other tumor types featuring NRF2 hyperactivation [145,146].

### 4.5. The Role of Fumarate in Regulation of KEAP1

One of the principal mechanisms that ensure NRF2 hyperactivation in RCC involves the destabilization of the binding between KEAP1 and NRF2, allowing for the nuclear translocation of the latter. Metabolic reprogramming is a common feature of RCCs and can be at the same time the cause and the effect of deregulation of pathways that trigger tumor progression, such as KEAP1-NRF2. PRCC type II features a defect in the production of fumarate hydratase (FH), a key enzyme of the tricarboxylic acid cycle (TCA), responsible for the conversion of fumarate into malate. This leads to the intracellular accumulation of fumarate, that has been described to support the progression of *FH*-deficient tumors, as occurs in both the hereditary (HLRCC) and sporadic (PRCC) forms of RCC [19,147]. Fumarate behaves as an oncometabolite, able to support tumor progression through different mechanisms. Indeed, it was described that fumarate accumulation drives EMT transformation in kidney epithelial cells, through the inhibition of miR200ba429, thus promoting renal carcinomas formation in both *FH* deficient HLRCC and *FH* proficient cells treated with exogenous fumarate [148]. In addition to epigenetic regulation, fumarate is responsible for stabilization of both hypoxia inducible factor (HIF)-1α and NRF2, two central drivers of RCC progression (Figure 2). In the first case, fumarate acts as a competitive inhibitor of the 2-oxoglutarate-dependent dehydrogenase, particularly the HIF-1α prolyl hydroxylases (PHDs), allowing VHL-dependent degradation. This axis accounts for stabilization of HIF-1α in normoxic conditions (pseudohypoxia), known to promote tumor formation, as described in ccRCC [149,150,151]. Moreover, fumarate accumulation is involved in a process called succination, in which there is nucleophilic addition of fumarate on cysteine residues (Michael addition type reaction) forming adducts of S-2-succynil cysteine (2SC), that have been proposed as marker for HLRCC and PRCC type II featuring *FH* mutations that are not detected as changes in the protein levels [152]. Through this mechanism, fumarate interacts with Cys151 and Cys288 in KEAP1, inducing the release of NRF2, which can translocate into the nucleus and support target genes production [153,154,155]. In 2011, it was demonstrated that NRF2 hyperactivation is a driving mechanism of tumor progression in PRCC featuring *FH* deficiency [20,66]. The cytoplasmic and nuclear accumulation of NRF2 occurs only in tumor cells and not in stroma and starts in the early stages of the disease, probably supporting the malignant transformation of cysts, whose formation is independent of HIF-1α [20,66]. Indeed, our group described a new murine model of PRCC type II, in which mice feature slowly progressive transformation of cystic epithelium into carcinomas, in consequence of inactivation of a single gene *Tsc1,* and mTORC1 hyperactivation. Interestingly, this associates with downregulation of *Fh1* expression, accumulation of fumarate, and hyperactivation of the NRF2 axis, likely supporting the malignant phenotype establishment [156]. This model suggests that the novel link between mTORC1 and FH, and the downstream NRF2 hyperactivation, is conserved among RCC types, in fact the cooccurrence between these events is reported in human specimens of ccRCC, as in PRCC type II [157]. This observation makes NRF2 an interesting potential target for the treatment of RCC.

In this perspective, it is interesting to go deeper in detail concerning role of fumarate in the regulation of the NRF2 axis. We have just described that fumarate accumulation triggers cancer progression possibly through the hyperactivation of NRF2; on the other hand, dimethyl fumarate (DMF), a cell permeable methyl ester of fumaric acid, has been approved by the FDA for the treatment of relapsing forms of multiple sclerosis and psoriasis in relation to its anti-inflammatory properties [32,158]. Indeed, DMF is in clinical trial for the treatment of different cancer types, considering its link with a wide range of pathways and kinases involved in tumor progression, such as the KEAP1-NRF2 pathway, NF-κB, ERK, and MAPKs [32]. What is particularly interestingly is that modulation of the concentration of DMF can differentially regulate the activation of NRF2 signaling. Indeed, Saidu et al. have reported that low doses of DMF in cancer cells promote activation of NRF2 antioxidant pathway, impairing KEAP1 binding and resulting in cytoprotective effects and tumor progression. On the other hand, high doses of DMF not only result in succination of KEAP1, but also DJ-1, an NRF2 binding protein encoded by the *PARK7* gene and necessary for NRF2 nuclear translocation. This modification on Cys106 accounts for a reduction of nuclear NRF2 and consequent tumor cell sensitization to cell death [31]. This effect is not evident in normal cells, where the expression of DJ-1 is very low, while it is further exacerbated in KRAS mutated cells, as some kind of RCC [31,159]. Initially, this pattern of NRF2 regulation seemed to be in contrast with what was described for *FH* deficient RCCs. However, we need to take into account the dose, distribution, and bioavailability of endogenous fumarate compared to the exogenous, cell-permeable compound. We also have to take into account that acute or chronic activation of the NRF2 axis have opposite effects, as described above, thus the timing of exposure to fumarate could be critical for the outcome in cancer cells. The third important consideration is that the intracellular compartmentalization of the accumulated fumarate might play a role. Indeed, FH has been extensively described as a mitochondrial metabolic enzyme of the TCA cycle, however, a second transcript exists that lacks the mitochondrial targeting sequence, thus resides in the cytoplasm and can shuttle into the nucleus in response to DNA damage, in which fumarate plays a crucial role [160,161]. Thus, it was proposed that the two mechanisms resulting from *FH* loss can cooperate for tumor progression. In a first stage, the inability to produce fumarate near the nucleus impairs DNA damage repair and accounts for accumulation of mutations, then the proliferation of *FH* deficient cells leads to accumulation of fumarate that supports tumor progression [162]. Indeed, it has been demonstrated that fumarate accumulation and effect through NRF2 does not depend on mitochondrial damage, thus the phenotype is corrected introducing an extramitochondrial form of *Fh1* in the presence of persistent defective mitochondrial oxidative metabolism [66,151]. All these observations taken together suggest that understanding the fine regulation of the *FH*-fumarate-NRF2 axis represents an important step towards designing a new therapeutic strategy for the treatment of tumors as RCC.

## 5. Conclusions and Highlights about Therapeutical Strategies

RCCs are a complex group of tumors that have long been classified only on the basis of histological characteristics. However, in the last years genomic and molecular drivers of the disease have been discovered, characterizing specific and common patterns that can be exploited for new therapeutical strategies. As previously reported, ccRCC features mutations mainly in VHL/HIF and PI3K/AKT/mTOR pathways [14], thus the currently approved therapeutical approaches involve VEGF or mTOR inhibitors (such as Tensirolimus or Everolimus), for advanced RCC. Despite the high efficacy of these treatments, the main problem remains the insurgency of progressive disease in almost all cases, with tumors developing resistance [30]. On the other hand, PRCC is mainly characterized by mutations in the *MET* gene or loss of the *FH* gene that characterize type I and type II, respectively [11]. Currently, these markers of disease have been exploited for the development of target therapies that underwent different clinical trials [163]. However, new studies aimed to target other aspects of PRCC, that are common with ccRCC, such as the metabolic rewiring, are opening new perspectives in therapeutic approaches for RCCs [30].

Alterations in NRF2 signature are one of the targets initially related to PRCC type II, but that is becoming a common feature among the principal RCC types. Indeed, it is reported to be tightly regulated by different pathways triggering RCC progression and to be involved in the promotion of tumor growth and resistance to therapy. Thus, KEAP1-NRF2 can represent a potential target for new combinatory therapeutic approaches in RCC. We extensively described that NRF2 acts as a kind of two-faced Janus in RCC and the surrounding kidney epithelium, as already reported for other tumors [34]. In fact, while a transitory activation of NRF2-ARE signature protects the renal epithelium from injury and stress stimuli, the constitutive hyperactivation of NRF2 in renal cancer supports its survival and malignant behavior. Unfortunately, currently there are not yet specific NRF2 inhibitors, probably due to the structural similarity with other proteins of the bZip family [164]. Thus, understanding the different pathways and players that cooperate in sustaining the aberrant activation of NRF2 in cancers such as RCC represents a good opportunity to identify indirect targeted strategies. Indeed, this strategy would allow the utilization of targeted drugs already approved for other pathological conditions. Panieri et al. recently reviewed the currently available strategies to indirectly modulate the KEAP1-NRF2 axis. Among them there are both natural and chemical compounds acting at different steps of NRF2 regulation [165]. Here, we focus our attention on compounds targeting pathways that are dysregulated in RCC and with reported activity in NRF2 regulation. A first class of natural compounds counteract the aberrant accumulation of the protein. Among them there are Halofuginone, AEM1, Brusatol that downregulate both NRF2 protein and target genes [8,165], even if Brusatol was pointed out to downregulate general mRNA translation and not directly NRF2 [166]. Moreover, epigallocatechin 3-gallate, that was previously described to activate Nrf2 counteracting kidney injury, at high doses induces apoptosis in adenocarcinoma cells, downregulating the constitutively active NRF2 and overcoming the acquired resistance [167]. Other compounds act on proteins that can interact with NRF2. For example, Oridonin, a promising therapy for different kinds of cancer, suppresses both NF-κB and NRF2, preventing its nuclear translocation in osteosarcoma cells [168], while Wogonin can decrease the binding of p65 to *NRF2* promoter, leading to tumor cells being more sensitive to chemotherapeutic drugs [169]. Indeed, a specific inhibitor of P-p62 and KEAP1 interaction has shown good results in HCC, through downregulation of NRF2 activation [145]. Moreover, NRF2 axis is tightly regulated by post-translational modifications, including phosphorylation, acetylation, and succination. Several pathways involved at this level of NRF2 modulation are dysregulated in RCC, as reported in the previous sections. Thus, specific inhibitors of this pathways can be exploited to indirectly counteract NRF2 aberrant activation. Among the principal targets there are PI3K/AKT and ERK pathways. The natural flavone Chrisin, interfering with these two pathways, decreased Nrf2 both at the mRNA and protein levels, sensitizing HCC cells to chemotherapy [170]. Moreover, LGK-974 WNT inhibitor impaired NRF2 nuclear translocation in HepG2 cells, through inhibition of GSK-3β-TrCP protein complex [171]. Interestingly, dimethyl fumarate, which is approved by FDA as an NRF2 activator, displays some anticancer activities, and indeed at high concentrations it appears to behave as a NRF2 inhibitor [32]. This potential new implication of the synthetic form of fumarate has still not been evaluated but suggests that fumarate modulation could be a strategy in counteracting different type of cancers. Indeed, an interesting study by Sourbier et al. suggested that a combinatory treatment targeting both the metabolic rewiring and the activation of NRF2-dependent antioxidant signature is effective in FH-deficient RCC. They showed that in RCC featuring fumarate accumulation, the activation of Abelson murine leukemia viral oncogene homolog 1 (ABL1) simultaneously triggers the transcription of glycolytic genes through mTOR-dependent HIF stabilization and supports NRF2-dependent antioxidant signature activation. Thus, they proposed a combinatory treatment with Vandetanib that inhibits ABL1-dependent activation of mTOR, and Metformin, promoting AMPK-dependent activation of SIRT1, ultimately inhibiting NRF2 nuclear translocation, as an effective treatment for FH-deficient RCC [22]. This study supports the idea that a combinatory targeted therapy, modulating NRF2-ARE signature directly or indirectly, can be a promising opportunity for the treatment of RCCs featuring NRF2 hyperactivation.

## Figures and Tables

**Table 1:** Mutations and alterations of principal NRF2 target genes in PRCC and ccRCC.

Function	Genes	Mutations and Alterations	
		PRCC	ccRCC
**Antioxidant response**	*HMOX1*	TM	MM
	*GPX1*	Del	MM
	*TXN*	MM	Del
**Drug metabolism and disposition**	*NQO1*	Amp	Amp
	*AKR1B10*	MM, Del	Amp
	*AKR1C1*	MM, Amp	MM
	*AKR1C3*	MM, Amp	-
**Autophagy**	*SQSTM1*	MM, Amp	Amp
**Mitochondrial apoptosis**	*PARK7*	Amp, Del	Del
**Xenobiotic response and metabolism**	*AHR*	MM	TM, Amp
**NADPH generation and pentose synthesis**	*G6PD*	Amp	MM, Amp
	*IDH1*	Del	MM, TM, Amp, Del

Mutations and alterations of NRF2 target genes were extracted through cBioPortal, analyzing samples in TCGA PanCancer Atlas. Alterations are indicated as missense mutations (MM), truncating mutations (TM), deletions (Del), amplifications (Amp), not mutated or altered (-).

**Table 2:** *NFE2L2* and *KEAP1* somatic mutations and copy number alterations identified in human ccRCC and PRCC.

*NFE2L2* mutations				*KEAP1* mutations			
**ccRCC**				**ccRCC**			
*CDS* mutation	*AA* mutation	Mutation	Ref.	*CDS* mutation	*AA* mutation	Mutation	Ref.
c.70T>C	p.W24R	MM	[76]	c.160T>G	p.Y54D	MM	[12]
c.85C>G	p.D29H	MM	[76] *TCGA*	c.761A>C	p.K254T	MM	[23]
c.86A>T	p.D29V	MM	[77]	c.779G>A	p.R260Q	MM	[23]
c.89T>A	p.L30H	MM	[12]	c.779G>T	p.R260L	MM	[23]
c.92G>C	p.G31A	MM	[77]	c.1226T>C	p.M409T	MM	[12]
c.100C>G	p.R34G	MM	[12]	c.1330G>T	p.E444	NsM	[23]
c.239C>A	p.T80K	MM	[23]	c.1630T>C	p.W544R	MM	[12]
c.242G>A	p.G81D	MM	[12]	c.1735G>T	p.D759Y	MM	[23]
c.246A>C	p.E82D	MM	[12]	c.1752del	p.Y584	Del	[23]
c.739C>T	p.L247F	MM	[12]				
c.1279G>T	p.E427	NsM	[12]				
**PRCC**				**PRCC**			
*CDS* mutation	*AA* mutation	Mutation	Ref.	*CDS* mutation	*AA* mutation	Mutation	Ref.
c.70T>C	p.W24R	MM	[78]	c.532C>T	p.Q178	NsM	*TCGA*
c.85G>T	p.D29Y	MM	*TCGA*				
c.88C>T	L30F	MM	[68]				
c.89T>G	p.L30R	MM	*TCGA*	**Copy number alterations**			
c.106_108del	p.V36del	Del	[72]	**ccRCC**			
c.230A>C	p.D77A	MM	[68]	Gene	Cytoband	Alteration	Ref.
c.239C>A	p.T80K	MM	*TCGA*	*NFE2L2*	2q31.2	Amp	[12]
c.242G>T	p.G81V	MM	*TCGA*	*KEAP1*	19p13.2	Del	[12]
c.245A>G	p.E82G	MM	[72] *TCGA*				
c.246A>C	p.E82D	MM	*TCGA*	**PRCC**			
	p.F339L	MM	*TCGA*	Gene	Cytoband	Alteration	Ref.
	p.P174T	MM	*TCGA*	*NFE2L2*	2q31.2	Amp	[12]

Mutations and alterations were extracted by COSMIC (Catalogue of Somatic Mutations in Cancer) database through cBioPortal and are listed with the relative reference. Alterations are indicated as missense mutations (MM), nonsense mutations (NsM), deletions (Del), amplifications (Amp).
